# The self-help app My Grief: Bereaved parents' experiences of helpfulness, satisfaction and usability

**DOI:** 10.1016/j.invent.2024.100712

**Published:** 2024-01-17

**Authors:** Rakel Eklund, Maarten C. Eisma, Paul A. Boelen, Filip K. Arnberg, Josefin Sveen

**Affiliations:** a Department of Women's and Children's Health, Uppsala University, Uppsala University Hospital, 751 85 Uppsala, Sweden; b Department of Clinical Psychology and Experimental Psychopathology, University of Groningen, Groningen, the Netherlands; c Department of Clinical Psychology, Utrecht University, Utrecht, the Netherlands; d ARQ National Psychotrauma Centre, Diemen, the Netherlands; e National Centre for Disaster Psychiatry, Department of Medical Sciences, Uppsala University, Uppsala University Hospital, 751 85 Uppsala, Sweden; f Centre for Crisis Psychology, University of Bergen, Postbox 7800, 5020 Bergen, Norway

**Keywords:** Bereavement, mHealth, Prolonged grief, Randomized controlled trial, User experience

## Abstract

Mobile health (mHealth) apps have been shown to be useful to monitor and reduce mental health problems across a variety of stress-related and affective disorders, yet research on the value of apps for prolonged grief is scarce. Therefore, the main aim of this study was to elucidate bereaved parents' experiences of using the self-help app My Grief with a focus on helpfulness, satisfaction, and usability. Data were derived from closed-ended and open-ended questions administered at the 3-month post-assessment of the intervention group (*n* = 67) within a randomized controlled trial testing the effects of access to the My Grief app. The sample consisted of 88 % women, with a mean age of 47 years, who predominantly lost their child to cancer (41 %), on average 4.8 years ago. Participating parents indicated that the My Grief app helped them increase their knowledge about prolonged grief and track their grief over time. The app was experienced as easy to navigate and around half of the parents used the app more than one day a week. Almost all parents were satisfied with the app and would recommend it to other parents in similar situations. The findings add to the knowledge base justifying mHealth within support systems for bereaved adults.

## Highlights

• My Grief is the first self-help app developed and tested for prolonged grief.
• Parents reported that the app increased knowledge about grief and prolonged grief.
• My Grief app was experienced as easy to navigate in.
• Parents would recommend the My Grief app to other parents in similar situations.
• Parents reported being satisfied with the app, that it was useful and helped them.

## 1 Introduction

The death of a child is one of the most disruptive events that a parent can experience. Child loss affects many aspects of the bereaved parents' life for many years, such as psychological and physical health, societal functioning, and spiritual well-being (Vig et al., 2021). Bereaved parents have increased psychiatric and medical admissions (Li et al., 2005) and increased mortality compared to parents who did not lose a child (Li et al., 2003; Rostila et al., 2012). The loss of a child has been associated with elevated symptoms of depression, anxiety (Kreicbergs et al., 2004), and post-traumatic stress, as well as severe, persistent, and disabling grief, termed prolonged grief (Pohlkamp et al., 2019; Pohlkamp et al., 2018). Previous research found that mothers reported more severe prolonged grief symptoms than fathers 1–5 years post-loss (Pohlkamp et al., 2019).

Effective interventions for prolonged grief have been developed, including face-to-face therapy and guided and unguided internet-based therapies delivered via a variety of modalities (Doering and Eisma, 2016). Particularly cognitive-behavior therapy (CBT) is effective in treating prolonged grief symptoms. In CBT for prolonged grief disorder (PGD), the development and persistence of symptoms associated with PGD are proposed to be the result of three core processes: 1) Insufficient integration of the loss within autobiographical memory, 2) negative beliefs about oneself, the world, and the future, and 3) anxious avoidance of reminders of the loss and depressive avoidance of activities that may foster recovery (Doering and Eisma, 2016; Boelen et al., 2006). Therefore, CBT-treatment for PGD consists of creating a coherent, meaningful narrative of oneself regarding the loss; challenging negative beliefs about the self, the world and the future; gradually confronting avoided reminders of the loss, such as places, memories, or objects, and setting new life goals and engaging in new meaningful activities (Doering and Eisma, 2016). Other studies have focused on using complementary therapeutic techniques, such as mindfulness and relaxation exercises, to alleviate prolonged grief (Knowles et al., 2021; Lenferink et al., 2019; O'Connor et al., 2014). CBT for bereaved adults with different types of losses (e.g., loss of a child, suicide) has been shown to be effective in reducing symptoms of prolonged grief, depression, and posttraumatic stress both face-to-face (Boelen et al., 2007; Bryant et al., 2014; Rosner et al., 2014; Shear et al., 2005) as well as in internet-based formats, with the latter yielding moderate to large effects (Eisma et al., 2015; Kersting et al., 2013; Treml et al., 2021; Reitsma et al., 2023, for reviews see: Wagner et al., 2020; Zuelke et al., 2021). Additionally, it has been demonstrated that user satisfaction with internet-based grief treatments is generally high, and are generally perceived to be of high quality (Zuelke et al., 2021).

Yet, research on mobile health interventions (mHealth) for prolonged grief is still very limited. This is surprising, because mHealth has been proven to provide useful tools, both feasible, acceptable, and effective, for a variety of psychiatric conditions such as bipolar disorder, posttraumatic stress disorder, alcohol abuse, and sleeping problems (Bush et al., 2019; Wang et al., 2018). It has been shown to be helpful in improving illness self-management, preventing relapse, promoting adherence to medication, delivering psychoeducation, supporting recovery, and symptom monitoring (Naslund et al., 2015). Additionally, mHealth offers advantages over both face-to-face and web-based interventions because it can potentially be easily accessed, provide immediate support, be anonymous, at a low cost for the user, and is tailored to users' needs (Olff, 2015). Therefore, mHealth has great potential to be used as a self-help intervention for mental health problems, such as prolonged grief.

One app that uniquely focuses on mental health problems following a major negative life event and aims to reduce mental health problems is the app PTSD Coach. This self-management app focuses on improving knowledge about PTSD symptoms and provides support for trauma-related distress (Kuhn et al., 2018). Several studies on PTSD Coach, performed around the world, show that participants experience no or very few adverse negative effects of using the app, and that using the app is both feasible and acceptable (Cernvall et al., 2018; Hensler et al., 2022; Miner et al., 2016). A randomized control trial conducted in Sweden showed a reduction of symptoms in PTSD and depression among people using the app, compared to people in a waitlist control group (Hensler et al., 2022).

**Fig. 1:**
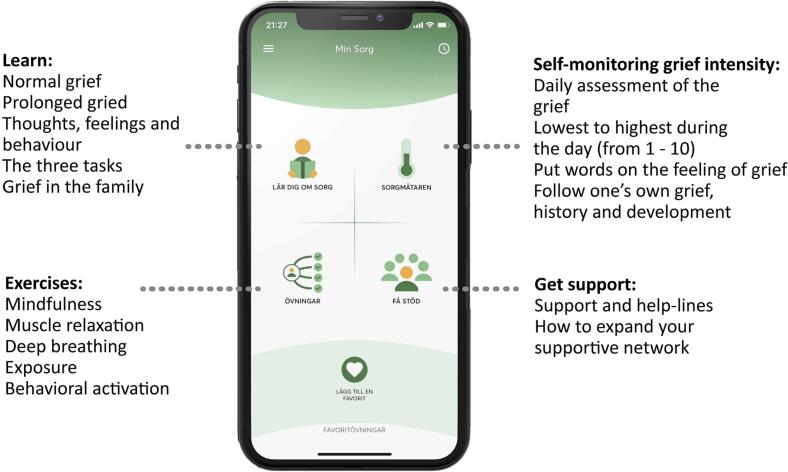
Structure of the My Grief app.

We have developed the first self-help app for prolonged grief, called My Grief (Swedish: Min Sorg). The app was developed for parents who have lost a child and is based on CBT for prolonged grief (Doering and Eisma, 2016; Boelen et al., 2006). My Grief uses the PTSD Coach structure and consists of four sections: 1) Learn – which includes psychoeducation regarding grief and prolonged grief, 2) Self-monitoring of grief intensity – a rating scale to monitor the daily intensity of grief, 3) Exercises – including mindfulness and relaxation techniques (via self-guided audio tracks) and exposure to memories associated with the loss (writing exercises) and 4) Get support – with contact information of different support functions, public services, psychoeducation about social networking, and information about other parents' experiences of losing a child (Fig. 1).

Prior to the current study, we conducted a mixed-method open trial feasibility study using pre-post surveys and a post-intervention interview (Eklund et al., 2022). Thirteen parents had access to the app My Grief for four weeks. Ten participants completed the follow-up survey, and eight completed the interview. It showed that using My Grief was feasible and acceptable for bereaved parents, as they reported it was easy to use, normalized grief reactions, and provided valuable knowledge and information. The study also showed that access to My Grief might reduce prolonged grief symptoms and maladaptive cognitive-behavioral processes in bereaved parents, with moderate to large pre- to post-intervention effect sizes (Eklund et al., 2022).

In the current study, we sought to further evaluate the app by deriving data from the intervention arm of a randomized controlled trial. The aim of this study was to elucidate bereaved parents' experiences of using the app My Grief. Specifically, we sought to clarify the app's perceived helpfulness, user satisfaction and usability.

## 2 Material and methods

### 2.1 Design

This paper focuses on evaluating the experiences of bereaved parents in the intervention group of a two-armed parallel-group randomized waitlist-controlled trial testing the effects of three months access to the app My Grief (Eklund et al., 2021).

### 2.2 Settings and participants

This study was approved by the Swedish Ethical Review Authority (project no. 2021–00770 and 2021-05333-02). Parents were recruited via a register and social media. Specifically, deceased children were identified via *the Swedish Childhood Cancer Registry*, which then was linked with *the Cause of Death Register* (children that died because of their cancer diagnosis were linked to the register). After receiving the social security numbers of the deceased children (*n* = 509), we contacted *the Swedish Tax Agency* to obtain information on the guardians (*n* = 972) and their addresses. Between September–November 2021, the identified parents received an information letter to their home address describing the study purpose. Additionally, we advertised the study via social media platforms and webpages from two non-profit organizations in Sweden for parents who lost a child during January 2022.

Inclusion criteria were a) being a parent of a child who died between 1 and 10 years ago, b) having mild to severe levels of prolonged grief [cut-off >16 on the Prolonged Grief Disorder-13 scale (Prigerson et al., 2009)], c) understanding and speaking Swedish, and d) having access to a smartphone. Exclusion criteria were self-reported current suicidal thoughts and/or a psychosis diagnosis. The parents signed up for the study via the project website: www.minsorg.com, which automatically directed the parents to a screening survey. Eligible parents received written information about the study and gave written informed consent to participate. Subsequently, they filled in the online pre-assessment survey at an online platform, RedCap, hosted by Uppsala University, Sweden.

**Fig. 2:**
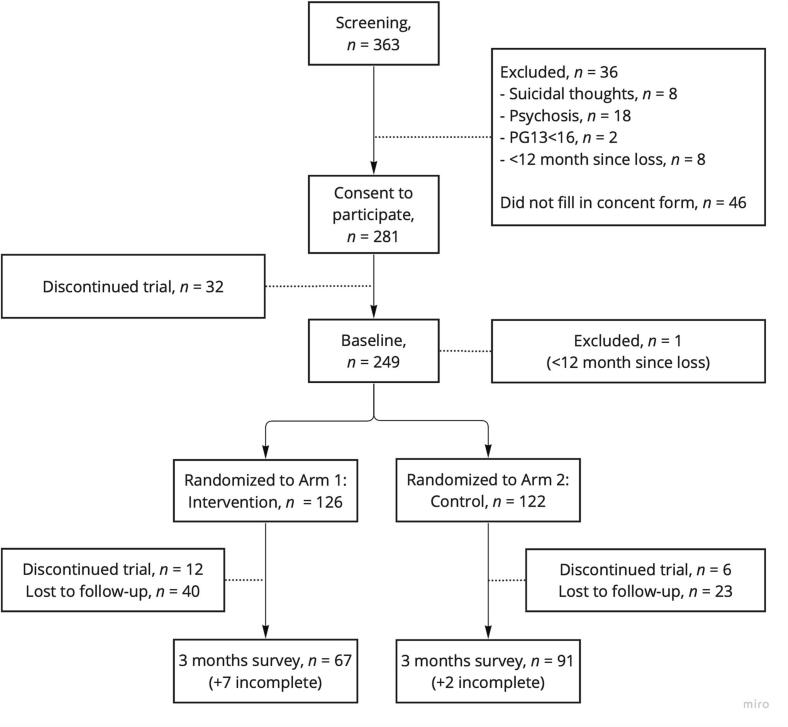
Flowchart over recruitment and pre-post assessments.

Of the 972 parents identified via the Swedish Childhood Cancer Registry, 151 parents (16 %) signed up for the study. Additionally, 202 parents recruited via social media signed up for the study. Of the 363 parents who signed up for the study, 248 met the criteria for inclusion and completed the online pre-assessment survey (Fig. 2). Parents were randomized to arm 1 (intervention group, *n* = 126) or arm 2 (waitlist, *n* = 122). Parents living together were manually allocated to the same arm as the other parent (*n* = 24). Parents allocated to the intervention group received an e-mail describing how to download the app, and received access to the app for three months. After three months, the participants received an e-mail with a link to the online post-assessment survey. A total of 67 (53 %) parents completed the follow-up survey. If the parents did not complete the survey, they received one reminder via e-mail and two reminders via text messages during a three-week period.

No significant differences between those who did (*n* = 67) and did not complete the post-assessment (*n* = 59) were found regarding time since loss of the child (*t*(121) = −1.8, *p* = .071), age of the parent (*t*(124) = −1.9, *p* = .053), or gender of the parent (*X*^2^(1, *N* = 126) = 3.6, *p* = .055). However, participants born in another country than Sweden were less likely to complete the post-assessment (*X*^2^(1, *N* = 126) =7.1, *p* = .008).

### 2.3 Measurements

The pre-assessment consisted of a questionnaire assessing sociodemographic characteristics (see Table 1) and loss-related characteristics (see Table 2). Additionally, instruments to assess symptoms of prolonged grief, depression, post-traumatic stress, and cognitive-behavioral variables were included in pre- and post-assessments but were not analyzed within the present study. The post-assessment for the intervention group included 32 questions (both closed-ended and open-ended) regarding the app's perceived helpfulness, user satisfaction, usability, and negative consequences of using the app.

**Table 1:** Sociodemographic characteristics of parents with app-access.

Characteristics	Parents, *n* = 67
Sex	*n* (%)
Female	59 (88)
Male	8 (12)
Age	Mean (*SD*) min–max 47.4 (10.9), 27–73
Place of residence	*n* (%)
Rural area	15 (22)
Small town	13 (19)
Suburb	7 (10)
Town	11 (16)
Larger town	21 (31)
Country of birth	*n* (%)
Sweden	66 (98)
Other	1 (2)
Completed education	*n* (%)
Elementary school	1 (2)
Senior high school	22 (33)
University	44 (66)
Occupation	*n* (%)
Employed, student	52 (78)
Parental leave, sick-leave, care for sick children, pensioner	15 (22)
Civil status	*n* (%)
Married or committed non-marriage relationship	60 (90)
Single	6 (8)
Widowed	1 (2)
Children within family (including those who died and children over 18 years)	*n* (%)
1	9 (13)
2	19 (28)
3	31 (46)
4+	8 (12)

**Table 2:** Loss-related characteristics.

Characteristics	Children, *n* = 63
Gender of deceased child	*n* (%)
Girl	25 (40)
Boy	37 (59)
Non-binary	1 (2)
Deceased child's age at time of death	*n* (%)
Stillborn (pregnancy week 20–38 or during labor)	11 (17)
Within the first year	7 (11)
1–6 years old	11 (17)
7–12 years old	5 (8)
13–19 years old	10 (16)
20–29 years old	16 (25)
30–39 years old	3 (5)
Cause of death	*n* (%)
Cancer	26 (41)
Stillbirth	11 (17)
Infant death (under 1 year)	5 (8)
Accident (e.g., traffic, fall, diving)	6 (9)
Mental health problems (e.g., suicide, overdose)	6 (9)
Other diseases (e.g., cardiac arrest, epilepsy, COVID-19)	7 (11)
Other causes (murder)	1 (2)
Unknown	1 (2)
Years since death (from date of death to baseline)	Mean (*SD*) range 4.8 (2.9), 1–15 years
Lost another child	*n* (%)
Yes	7 (10)
No	60 (90)

We measured perceived helpfulness with ten items from the PTSD Coach app survey (Kuhn et al., 2014). The questionnaire was revised to fit the purposes of the current study by changing the diagnosis from PTSD to PGD and excluding four questions regarding stigmatization, myths, and treatments. These items were rated on a 5-point scale (from 1 “not at all” to 5 “extremely”). Additionally, two study-specific open-ended questions were included, namely: “Which sections did you use most frequently?” and “Which sections did you find most helpful?” User satisfaction was measured with three study-specific closed-ended questions about overall satisfaction (from 0 “not at all” to 3 “very”). We asked if the app met expectations and if participants would recommend the app to other parents (answer options: “yes” or “no”). Parents that did not recommend the app were asked to state the reason in a free-text response. Further, four study-specific open questions were included to establish which part of the app participants liked the least and most, if anything was missing in the app, and to probe any thoughts participants had about the name of the app. App usability was measured with two study-specific closed-ended questions, namely “Which sections have you used?” and “How often did you use the app?”. In addition, three study-specific open-ended questions were included to establish if the participants had encountered technical problems, what they thought about the app layout, and what possible improvements could be made to the app.

### 2.4 Data analysis

Data were derived from a survey that included both closed-ended and open-ended questions. Closed-ended questions were analyzed in SPSS (version 28, IBM, 2021) using descriptive statistics. Free-text responses were analyzed using summative content analysis (Hsieh and Shannon, 2005). The answers to the open questions were often short, from one word up to one sentence, e.g., mentioning different parts of the app or expressing a feeling with one word. The first and the last author read all free-text answers. The first author then counted and sorted the answers under every question into themes based on differences or similarities in content.

## 3 Results

### 3.1 Participants characteristics

A majority of the participants (*n* = 59, 88 %) were female, with a mean age of 47.4 years (range: 27–73, *SD*: 10.9) (Table 1). See Table 1 for sample characteristics and Table 2 for loss-related characteristics. Almost two-thirds (*n* = 43, 64 %) used iOS as their mobile operative system.

### 3.2 Helpfulness

The app My Grief was reported to be helpful by parents in many ways. For example, two-thirds (*n* = 47, 70 %) of the parents found the app moderately to extremely helpful in increasing their understanding of their grief reactions and their knowledge of prolonged grief. Around two-thirds, (*n* = 46, 69 %) of the parents also found that the app helped them feel that there was something they could do about their grief. Three in five parents (*n* = 40, 60 %) also rated the app moderately to extremely helpful in making them feel comfortable in seeking more help and support, and for tracking and following their grief over time (*n* = 44, 66 %) (Table 3).

**Table 3:** Perceived helpfulness of My Grief app.

Bereaved parents, *n* = 67	Not at all	Slightly	Moderately	Very	Extremely	Mean 1–5 (*SD*)
	*n* (%)	*n* (%)	*n* (%)	*n* (%)	*n* (%)	
Helping me learn about symptoms of prolonged grief	6 (9)	20 (30)	23 (34)	16 (24)	2 (3)	2.2 (0.99)
Helping me finding effective ways of managing my grief^a^	8 (12)	18 (27)	17 (25)	21 (31)	1 (2)	2.83 (1.06)
Helping me feel more comfortable in seeking help and support	17 (25)	10 (15)	27 (40)	10 (15)	3 (5)	2.58 (1.15)
Helping me feel there is something I can do about my grief	9 (13)	12 (18)	17 (25)	23 (34)	6 (9)	3.07 (1.19)
Helping me track my grief over time	9 (13)	14 (21)	20 (30)	17 (25)	7 (10)	2.99 (1.20)
Helping me know when I'm doing better or when I'm doing worse	8 (12)	14 (21)	24 (36)	18 (27)	3 (5)	2.91 (1.06)
Providing practical solutions to the problems I experience	13 (19)	18 (27)	18 (27)	14 (21)	4 (6)	2.67 (1.18)
Helping me better understand my grief reactions	9 (13)	11 (16)	23 (34)	18 (27)	6 (9)	3.01 (1.16)
Enhancing my knowledge of prolonged grief	13 (19)	8 (12)	19 (28)	19 (28)	8 (12)	3.01 (1.29)
Providing a way for me to talk about what I have been experiencing^b^	12 (18)	18 (27)	23 (34)	11 (16)	2 (3)	2.59 (1.06)

a *n* = 65.

b *n* = 66.

The answers to the open question further elucidated how the app helped the parents learn about their grief process, as one parent wrote: “*To help me better understand fluctuations in my mood. For example, sometimes I've felt totally infirm, but when I've taken the time to use the app, I can see that it's often around anniversaries and holidays. It makes it easier for me to be kind to myself and slow down. To give myself time to remember and talk.*” Furthermore, the parents found it helpful that they could choose when and how to use the app and appreciated the possibility to close it and open it again later.

When asked which sections they appreciate the most, all sections in the app were mentioned about equally often. The section “learn” was considered interesting to read, and participants found the provided information recognizable: “*Very good to read about grief and how it can be experienced and change over time*.” The “self-monitoring of grief intensity” increased the awareness of thoughts and feelings, helping the parents follow their grief process over time: “*It clearly shows that one's mood goes up and down; it became clear that my thoughts were always about the same thing*.” Even though many parents mentioned the self-monitoring section as one of the most helpful tools, some parents expressed the opposite as it could be challenging to rate one's feelings: “*Not the right tool for me, difficult to rate*.” The section “exercises” were considered helpful in coping with a lot of different feelings and the relaxation exercises were considered especially helpful: “*The exercises help me reduce anxiety and other negative emotions*.” The section regarding “getting support” was mentioned the least (Table 4). However, tips on podcasts and literature were appreciated most in this section. In response to the question regarding what was considered the most helpful about the app overall, one parent answered: “*That I have started to think more about my grief and what I need to do to help myself*.”

**Table 4:** User satisfaction and usability of My Grief app.

	Parents, *n* = 67
Overall, how satisfied are you with My Grief?	*n* (%)
Not at all	3 (5)
Indifferent/some	13 (19)
Moderate	19 (28)
Very	32 (48)
Would you recommend My Grief to other parents in similar situations?	*n* (%)
Yes	62 (92)
Did the app meet the expectations you had before you started using it?	*n* (%)
Yes	57 (85)
How often did you use the app?	*n* (%)
Every day	1 (2)
A few times a week	18 (27)
Once a week	9 (13)
Less than once a week	35 (52)
I did not use the app	3 (5)
Missing	1 (2)
The app consists of four sections. Which have you used? (multiple options are possible)	*n* (%)
Learn	53 (79)
Self-monitoring grief intensity	53 (79)
Exercises	43 (64)
Get support	22 (33)

### 3.3 User satisfaction

Overall, the parents were satisfied with the app, and almost all parents (*n* = 62, 92 %) would recommend the app to parents in similar situations (Table 3, Table 4). The five parents who did not recommend the app explained that they already had much knowledge regarding their grief or that it would have been better if it was possible to use the app together with other bereaved parents. Some also expressed that it would probably have been better to have the app at the beginning, the first year of the grieving process, and not many years after the loss: “*Would recommend it during the first year of grief or if you are really stuck in your grief*.” A majority of the parents (*n* = 57, 85 %) felt that the app lived up to the expectations that they had before using it.

A few parents expressed that they missed a feature where the parents could connect with each other within the app. Additionally, it was suggested that the app should be available earlier in the grieving process.

The name of the app, My Grief [In Swedish: Min Sorg] was appreciated by almost all parents, as many parents wrote similar things, for example one parent wrote: “*Good, because that's exactly how it is, my grief and no one else's*.” However, one parent did not appreciate the name, and wrote: “*Strange, too intimate*.”

### 3.4 Usability

Almost half of the parents stated that they used the app once a week or more (*n* = 28, 42 %), whereas the other half used it less than once a week (*n* = 35, 52 %) (Table 4). Some parents commented that they did not use the app as much as they intended: “*It is difficult to manage the information in the app while working and having other things in life, I haven't used it as often as I thought I would*”. All sections in the app were used by the parents (Table 4); most of the parents (*n* = 53, 79 %) used the sections “learn” and “self-monitoring” at least once.

The majority of the parents experienced the app's colors and icons as pleasant. They also indicated that it was easy to navigate the app and the layout was clear. One parent wrote: “*Very user-friendly. Calm and pleasant colors, good size of icons and clarity. It's an easy app to use and you quickly find the parts you use most. Especially like the little spinning wheel where you can save your favorite exercises for quick access, e.g., a breathing exercise*.” Two parents expressed that their grief made them tired, and it was hard to process the information; therefore, the amount of text in the app was considered too much to handle and the app felt a bit messy.

Furthermore, the parents in this study had some suggestions on how to improve the app. For example, the parents wanted to do the writing exercises directly in the app and not have to write on paper. They also wanted a possibility to connect with other parents via the app, receive reminders with comforting sentences, and tips on where to find lectures about grief around the country. Further, they suggested that the app should be available early in the grieving process and not just for parents with prolonged grief.

During the three-month testing period, a few parents (*n* = 10, 15 %) reported that the app had some minor bugs, such as crashing when opening it from the app reminders. Two of the parents had trouble with installing the app but not when using it later on. Overall, there were very few technical issues reported.

## 4 Discussion

This study is the first comprehensive evaluation of bereaved parent's experiences with a self-help app for prolonged grief. The specific aim of the study was to elucidate bereaved parent's experience of the My Grief app, with a particular focus on the app's helpfulness, the user's satisfaction, and app usability.

Parents found the My Grief app to be helpful in various ways. They mentioned that it increased their knowledge about grief, allowed them to track their grief over time, and provided a sense of security and support. These results align with findings from our previous smaller feasibility trial, which also showed that parents reported increased knowledge about grief after using the app for four weeks (Eklund et al., 2022). However, the current study additionally shows that access to My Grief adds to the understanding of one's own grief reactions and provides a way to talk about previous experiences related to grief. The “learn section” of the app, which provided psychoeducation about grief, was judged favorably by the parents, which is also in line with our previous research (Eklund et al., 2022). Some parents perceived it as helpful that the app gave the participants the freedom to choose when and how to use the app, which is one major advantage of mHealth (Bush et al., 2019; Wang et al., 2018). As there are no studies evaluating apps for prolonged grief in adults, the extensive research conducted on the app PTSD Coach can be used to contextualize the findings regarding My Grief. Users highly valued the convenience of assessing My Grief whenever necessary, which is mirrored by findings on the PTSD Coach app (Owen et al., 2015).

In this study, we found high levels of satisfaction with the My Grief app as a whole, as well as many individual components of the app. Eighty-five percent of participants felt the app met their expectations and nearly all would recommend it to other parents in similar situations. This finding corresponds with results from previous research on the My Grief app and the related PTSD Coach app (Eklund et al., 2022; Kuhn et al., 2014).

Regarding usability, our findings indicate a dual user pattern of engagement with the app. Either participants used the app once a week or more, or they used it less than once a week. Participants using the app less than once a week often expressed that their usage of the app fell short of their initial intentions. However, participants in this study were told that they could use the app as much or as little as they wanted over a 3-month period. These patterns of interaction with mHealth tools are common and have extensively been discussed in previous research (Cernvall et al., 2018; Peng et al., 2016; Ben-Zeev et al., 2013). Owen and colleagues assessed user retention of the PTSD Coach app, using mobile analytic data, and they showed how the time spent on the app slowly declined over time, with 41.6 % continuing using the app one month after installation, to 10.6 % using the app one year post installation (Owen et al., 2015). However, the fact that the mHealth tools are used less over time is not necessarily negative, as they are designed to improve well-being. Decreased app usage could indicate that the person is feeling better and thus experiences a reduced need to use the app.

Moreover, our research findings indicate that My Grief is user-friendly, as the majority of the participants found it easy to navigate. Only two parents reported that they found it difficult to comprehend the app or indicated that they felt the app provided too much information. Previous research shows that it is important to develop an easy-to-follow interface and navigation in mHealth tools for persons with mental health problems. Additionally, app content should be easily accessible and the app content and interface should not be not too abstract (Ben-Zeev et al., 2013). My Grief was developed keeping in mind that bereaved people, especially those with severe grief reactions, often have difficulty assimilating large amounts of information and maintaining focus and concentration. For example, psychoeducation texts were kept short and several exercises were audio recorded.

This first comprehensive evaluation of an app for prolonged grief has offered unique insights. However, it also has some limitations. First, the sample was very homogenous, with an overrepresentation of highly educated women born in Sweden. This suggests that further evaluations may be needed to determine the acceptability of My Grief in other demographic groups, such as fathers who have lost a child, as well as within various cultural and socioeconomic groups in society. A second limitation of this study is that the evaluation is based on the self-reported opinions of the app rather than the effects of the app on mental health. A third limitation is the fairly high percentage of dropouts (47 %) in the study from pre-assessment to post-assessment, which may have led to a more favorable evaluation, as those who dropped out may have disliked certain features of the app but did not complete the post-assessment. Future mHealth trials may explore usability as a cause for dropout. Another reason for dropout, however, may be time constraints, because many bereaved parents have other children to care for. We further observed that people who were not born in Sweden were more likely to drop out, which may pose a threat to external validity.

Despite these limitations, the findings of this study highlight multiple beneficial aspects of the My Grief app, such as a high user satisfaction and a user-friendly interface. As many self-help apps in app stores are commercial and very few have validated evidence of effectiveness, the extensive evaluation of an mHealth tool such as My Grief offered by this study is a strength. Apps that are not validated, developed by companies rather than mental health professionals, could be unhelpful or cause harm to the user (Olff, 2015). Based on the findings from this study, as well as the results from our pilot trial (Eklund et al., 2022), the My Grief app is now available in app stores, free to download and use. However, the app is currently only available in Swedish, and we recommend translating the app in other languages and examining the app's effects in other countries. We note in this context that the promising results from the RCT assessing the effects of the app on psychopathology levels (Sveen et al., 2023) will be presented in other papers.

## 5 Conclusions

We have comprehensively tested the helpfulness, user satisfaction, and usability of the first app for prolonged grief in adults. Parents reported being satisfied with the app and that it was both helpful and easy to use. The present study adds to an evidentiary base that could help justify the implementation of mHealth for bereaved adults within healthcare and in other support systems. Overall, these findings provide a foundation for further research, including examining the potential effect of app usage on mental health outcomes, such as prolonged grief.

## Ethical approval

The study has received ethical approval from the Swedish Ethical Review Authority (no. 2021-00770; no. 2021-05333-02) and has been carried out in accordance with The Code of Ethics of the World Medical Association (Declaration of Helsinki). Informed consent was obtained from all participants.

## Declaration of competing interest

None.
